# Tópicos Emergentes em Insuficiência Cardíaca: Terapias Intervencionistas na Insuficiência Cardíaca

**DOI:** 10.36660/abc.20201086

**Published:** 2020-11-01

**Authors:** João Manoel Rossi, Dirceu Rodrigues de Almeida, Fernando Antibas Atik, Monica Samuel Avila, Marcely Gimenes Bonatto

**Affiliations:** 1 Instituto Dante Pazzanese de Cardiologia São PauloSP Brasil Instituto Dante Pazzanese de Cardiologia, São Paulo, SP – Brasil; 2 Universidade Federal de São Paulo São PauloSP Brasil Universidade Federal de São Paulo, São Paulo, SP - Brasil; 3 Universidade de Brasília BrasíliaDF Brasil Universidade de Brasília, Brasília, DF - Brasil; 4 Universidade de São Paulo Instituto do Coração do Hospital das Clínicas São PauloSP Brasil Instituto do Coração do Hospital das Clínicas da Universidade de São Paulo, São Paulo, SP – Brasil; 5 Hospital Santa Casa de Misericórdia de Curitiba CuritibaPR Brasil Hospital Santa Casa de Misericórdia de Curitiba, Curitiba, PR - Brasil

**Keywords:** Insuficiência da Valva Mitral, Marcapasso Cardíaco Artificial, Ablação por Cateter, Desfibriladores Implantáveis, Insuficiência da Valva Tricúspide

## Tratamento da Insuficiência Mitral Secundária

Antes da consideração do tratamento percutâneo da insuficiência mitral (IM) para pacientes com insuficiência cardíaca (IC) com fração de ejeção reduzida (ICFEr) e IM grave, ^1^ recomendamos que a terapia guiada por diretrizes esteja otimizada, incluindo terapia de ressincronização cardíaca e revascularização, quando apropriado.

O uso do dispositivo edge-to-edge poderia beneficiar pacientes com IM secundária moderadamente grave ou grave (área efetiva do orifício regurgitante [AEOR] ≥ 30 mm^2^ e/ou volume regurgitante > 45 mL) com fração de ejeção do ventrículo esquerdo (FEVE) de 20 a 50%, diâmetro sistólico final do ventrículo esquerdo (VE) < 7,0 cm e sintomas persistentes, apesar da terapia baseada em evidências maximizada, com a participação de equipe multidisciplinar experiente na avaliação e no tratamento da IC e IM. ^2^

O COAPT incluiu pacientes com IM mais grave e doença do VE menos avançada (dilatação/disfunção) em comparação aos pacientes do MITRA-FR, criando o conceito de IM desproporcional ( Tabela 1 ).

**Tabela 1:** Comparação das caraterísticas e resultados do estudo COAPT e MITRA-FR

	MITRA-FR	COAPT
**Total de pacientes**	304	**614**
**Terapia médica**	Ajustada no estudo	**Terapia otimizada**
**≥ moderada a importante (3+) IMi**	AEOR > 20 mm2 e/ou VR > 30 mL	**AEOR > 30 mm2 e/ou** **VR > 45 mL**
**DSFVE, mm (tamanho VE)**	Sem limite	**< 70 na inclusão**
**Classe NYHA > II, %**	**67,0**	60,4
**Hospital em anos anteriores, %**	**100**	57,1
**AEOR, mm2**	31 ± 10	41 ± 15
**Importante (AEOR ≥ 40 mm2), %**	16	**41**
**VDFVEi, mL/m2**	135 ± 35	**101 ± 34**
**Mortalidade em 1 ano (IvsC), %**	24,2 vs 22,4	**19,1 vs 23,2 (p < 0,001)**
**Hospitalização IC em 1 ano (IvsC), %**	48,7 vs 47,4	**35,8 vs 67,9 (p < 0,001) Objetivo primário**
**Mortalidade anual/ hospitalização IC (IvsC), %**	54,6 vs 51,3 (p = 0,53) Objetivo primário	**33,9 vs 46,5 (< 0,001)**

IMi: insuficiência mitral isquêmica; AEOR: área efetiva do orifício regurgitante; VR: volume regurgitante; DSFVE: diâmetro sistólico final do ventrículo esquerdo; NYHA: New York Heart Association; VDFVEi: índice volume diastólico final do ventrículo esquerdo; IvsC: Invasivo vs Controle; IC: insuficiência cardíaca.

## Quando Indicar o Cardioversor Desfibrilador Implantável (CDI) frente aos Novos Medicamentos na ICFEr?

### Miocardiopatia Isquêmica

Os estudos randomizados MADIT II e SCD-HeFT, ^3^ ^,^ ^4^ conduzidos há mais de 15 anos, validaram a indicação de CDIs para a prevenção primária de morte súbita (MS) em pacientes com miocardiopatia de etiologia isquêmica, com fração de ejeção ≤ 35%, em classe funcional II e III, após a otimização do tratamento clinico, com pelo menos 40 dias após a fase aguda do infarto do miocárdio e pelo menos 90 dias após algum procedimento de revascularização miocárdica, sem comorbidades graves e com boa expectativa de vida em um ano. Esses estudos foram conduzidos em uma época na qual o tratamento farmacológico estava muito aquém do desejável em termos de doses; atualmente, os medicamentos podem promover uma redução substancial na taxa anual de MS. ^5^ ^,^ ^6^

### Miocardiopatia não Isquêmica

Pequenos estudos randomizados (CAT, AMIOVIRT e DEFINITE), conduzidos há mais de 10 anos, não foram capazes de demonstrar redução de mortalidade com o emprego do CDI para a prevenção primária de MS na miocardiopatia não isquêmica. ^5^ Recentemente, o estudo DANISH ^7^ com casuística robusta e em pacientes bem tratados também demonstrou que o (CDI) não reduziu a mortalidade total ou a morte cardiovascular nessa população. Devemos considerar uma maior estratificação desses pacientes, incorporando a quantificação de fibrose na ressonância magnética, que tem demonstrado relação com mortalidade cardiovascular e MS em portadores de miocardiopatia não isquêmica. ^8^

## Isolamento de Veias Pulmonares no Tratamento da Fibrilação Atrial (FA) no Paciente com ICFEr

A ablação de FA em pacientes com IC tem um benefício maior do que o uso de drogas antiarrítmicas, pela maior taxa de manutenção de ritmo sinusal, melhora de capacidade funcional e qualidade de vida, NYHA, distância no teste de caminhada de 6 minutos, VO2 máximo, ^9^ redução de biomarcadores (BNP), aumento de fração de ejeção, ^9^ ^,^ ^10^ redução de hospitalização por IC, morte ou hospitalização por IC e morte por qualquer causa. ^9^ ^–^ ^11^ Entretanto, a taxa de sucesso varia de 60 a 80% no primeiro ano em que doença cardíaca estrutural é um fator de risco para recorrência. ^12^ O isolamento das veias pulmonares pode ser feito por radiofrequência ou crioablação e essas técnicas podem ser combinadas com ablação de outros substratos. Os benefícios poderiam ser o controle de sintomas em pacientes com FA paroxística/persistente ou a promoção do remodelamento reverso em pacientes com disfunção ventricular por taquicardiomiopatia induzida pela FA, independentemente dos sintomas.

## Novas Formas de Estimulação Cardíaca na IC

A estimulação ventricular através do sistema de condução nativo do feixe His-Purkinje poderia ser uma alternativa para pacientes com indicação de marcapasso, visto os efeitos deletérios da estimulação isolada do ventrículo direito (VD) em pacientes com IC. ^13^ Pequenos estudos sugerem que a estimulação hissiana pode resultar em menor incidência de cardiomiopatia, menor combinado de hospitalizações e óbito, melhora das dimensões do VE e sintomas de IC em relação à estimulação isolada do VD. ^13^ ^,^ ^14^ A diretriz americana de manejo de bradicardia recomenda a estimulação hissiana ou a terapia de ressincronização cardíaca para pacientes com disfunção ventricular que apresentarem bloqueio atrioventricular com indicação de marcapasso permanente no lugar da estimulação isolada de VD. ^15^ Quando comparada à terapia de ressincronização cardíaca, a estimulação do feixe de His demonstrou um efeito equivalente, com redução significante do complexo QRS, melhora da FEVE, dos sintomas de IC e da qualidade de vida. ^16^ ^,^ ^17^ A estimulação hissiana também foi testada em pacientes com ICFEr e bloqueio de ramo direito com aumento da FEVE e estreitamento do complexo QRS. ^18^

## Tratamento Percutâneo da Insuficiência Tricúspide na IC

O advento do tratamento percutâneo da insuficiência tricúspide funcional é atrativo em pacientes selecionados de alto risco cirúrgico, na expectativa de proporcionar melhora dos sintomas. Os pacientes que poderiam se beneficiar desse tratamento são aqueles com IC refratária ao tratamento clínico otimizado ou com sinais iniciais de disfunção ventricular direita, considerados de alto risco para cirurgias cardíacas convencionais ou inoperáveis. Os dispositivos são divididos em sistemas de anuloplastia, sistemas de reparo das cúspides e o implante de *stents* valvulados nas veias cavas. Embora os estudos clínicos em relação à segurança e à eficácia pareçam ser promissores, as evidências disponíveis na literatura são baseadas em estudos observacionais unicêntricos ou registros, sendo necessária evidências mais robustas para qualquer indicação terapêutica. ^19^

## Lista de Participantes do Heart Failure Summit Brazil 2020 / Departamento de Insuficiência Cardíaca - DEIC/SBC

Aguinaldo Freitas Junior, Andréia Biolo, Antonio Carlos Pereira Barretto, Antônio Lagoeiro Jorge, Bruno Biselli. Carlos Eduardo Montenegro, Denilson Campos de Albuquerque, Dirceu Rodrigues de Almeida, Edimar Alcides Bocchi, Edval Gomes dos Santos Júnior, Estêvão Lanna Figueiredo, Evandro Tinoco Mesquita, Fabiana G. Marcondes-Braga, Fábio Fernandes, Fabio Serra Silveira, Felix José Alvarez Ramires, Fernando Atik, Fernando Bacal, Flávio de Souza Brito, Germano Emilio Conceição Souza, Gustavo Calado de Aguiar Ribeiro, Humberto Villacorta Jr., Jefferson Luis Vieira, João David de Souza Neto, João Manoel Rossi Neto, José Albuquerque de Figueiredo Neto, Lídia Ana Zytynski Moura, Livia Adams Goldraich, Luís Beck-da-Silva Neto, Luís Eduardo Paim Rohde, Luiz Claudio Danzmann, Manoel Fernandes Canesin, Marcelo Bittencourt, Marcelo Westerlund Montera, Marcely Gimenes Bonatto, Marcus Vinicius Simões, Maria da Consolação Vieira Moreira, Miguel Morita Fernandes da Silva, Monica Samuel Avila, Mucio Tavares de Oliveira Junior, Nadine Clausell, Odilson Marcos Silvestre, Otavio Rizzi Coelho Filho, Pedro Vellosa Schwartzmann, Reinaldo Bulgarelli Bestetti, Ricardo Mourilhe Rocha, Sabrina Bernadez Pereira, Salvador Rassi, Sandrigo Mangini, Silvia Marinho Martins, Silvia Moreira Ayub Ferreira, Victor Sarli Issa.
