# Supplementary material for: Tópicos Emergentes em Insuficiência Cardíaca: Terapias Intervencionistas na Insuficiência Cardíaca
Source: Arq Bras Cardiol. 2020 Nov 1;115(5):953–5. [Article in Portuguese] doi: 10.36660/abc.20201086 (PMC8452214; doi:10.36660/abc.20201086)
Supplement: Translation

# Translation: Emerging Topics in Heart Failure: Interventional Heart Failure Therapies

**Authors:** João Manoel Rossi, Dirceu Rodrigues de Almeida, Fernando Antibas Atik, Monica Samuel Avila, Marcely Gimenes Bonatto

**Affiliations:** 1 São PauloSP Brazil Instituto Dante Pazzanese de Cardiologia, São Paulo, SP – Brazil; 2 São PauloSP Brazil Universidade Federal de São Paulo, São Paulo, SP - Brazil; 3 BrasíliaDF Brazil Universidade de Brasília, Brasília, DF - Brazil; 4 São PauloSP Brazil Instituto do Coração do Hospital das Clínicas da Universidade de São Paulo, São Paulo, SP – Brazil; 5 CuritibaPR Brazil Hospital Santa Casa de Misericórdia de Curitiba, Curitiba, PR - Brazil

**Keywords:** Mitral valve insufficiency, Pacemaker, Artificial, Catheter ablation, Defibrillators, Implantable, Tricuspid valve insufficiency

## Treatment of Secondary Mitral Regurgitation

Before considering percutaneous treatment of mitral regurgitation (MR) for patients with heart failure (HF) with reduced ejection fraction (HFrEF) and severe MR, ^1^ we recommend that guideline-directed medical therapy be optimized, including cardiac resynchronization therapy and revascularization, where appropriate.

The use of the edge-to-edge device could benefit patients with moderately severe or severe secondary MR (effective regurgitant orifice area [EROA] ≥ 30 mm^2^and/or regurgitant volume > 45 mL) with a left ventricular ejection fraction (LVEF) of 20 to 50%, left ventricular (LV) end-systolic diameter < 7.0 cm, and persistent symptoms despite maximized evidence-based medical therapy, with the participation of an experienced multidisciplinary team in the evaluation and treatment of HF and MR. ^2^

The COAPT trial included patients with more severe MR and less advanced LV disease (dilatation/dysfunction) compared to patients of the MITRA-FR trial, creating the concept of disproportionate MR ( Table 1 ).

**Table 1:** Comparison of the characteristics and results of the COAPT and MITRA-FR trials

	MITRA-FR	COAPT
**Total number of patients**	304	**614**
**Medical therapy**	Adjusted in the trial	**Optimized therapy**
**≥ moderate to severe (3+) iMR**	EROA > 20 mm2 and/or RV > 30 mL	**EROA > 30 mm2 and/or RV > 45 mL**
**LVESD, mm (LV size)**	Without limit	**< 70 upon inclusion**
**NYHA class > II, %**	**67.0**	60.4
**Hospital in previous years, %**	**100**	57.1
**EROA, mm2**	31 ± 10	**41 ± 15**
**Severe (EROA ≥ 40 mm2), %**	16	**41**
**iLVEDV, mL/m2**	135 ± 35	**101 ± 34**
**1-year mortality (IvsC), %**	24.2 ± 22.4	**19.1 vs 23.2 (p < 0.001)**
**1-year HF hospitalization (IvsC), %**	48.7 ± 47.4	**35.8 vs 67.9 (p < 0.001) Primary objective**
**Annual mortality/ HF hospitalization (IvsC), %**	54.6 vs 51.3 (p = 0.53) Primary objective	**33.9 vs 46.5 (p < 0.001)**

iMR: ischemic mitral regurgitation; EROA: effective regurgitant orifice area; RV: regurgitant volume; LVESD: left ventricular end-systolic diameter; NYHA: New York Heart Association; iLVEDV: indexed left ventricular end-diastolic volume; IvsC: Invasive vs Control; HF: heart failure.

## When to Indicate an Implantable Cardioverter Defibrillator (ICD) in Face of New Medications in HFrEF?

### Ischemic Cardiomyopathy

The randomized MADIT II and SCD-HeFT trials, ^3^ ^,^ ^4^ conducted more than 15 years ago, validated the indication of ICDs for the primary prevention of sudden cardiac death (SCD) in patients with ischemic cardiomyopathy with an ejection fraction ≤ 35% in New York Heart Association (NYHA) class II or III after optimization of medical therapy, after at least 40 days of the acute phase of myocardial infarction and at least 90 days of any myocardial revascularization procedure, without severe comorbidities and with good 1-year life expectancy. These trials were conducted at a time when pharmacologic treatment was far less than desirable in terms of doses. Currently, medications can promote a substantial reduction in the annual rate of SCD. ^5^ ^,^ ^6^

### Nonischemic Cardiomyopathy

Small randomized trials (CAT, AMIOVIRT, and DEFINITE), conducted more than 10 years ago, were unable to demonstrate a reduction in mortality with the use of ICDs for primary prevention of SCD in nonischemic cardiomyopathy. ^5^ Recently, the DANISH trial, ^7^ with a robust sample of properly treated patients, also demonstrated that ICDs did not reduce total mortality or cardiovascular death in this population. We should consider greater risk stratification in these patients by incorporating magnetic resonance imaging quantification of fibrosis, which has shown to be associated with cardiovascular death and SCD in patients with nonischemic cardiomyopathy. ^8^

## Pulmonary-vein Isolation for the Treatment of Atrial Fibrillation (AF) in Patients with HFrEF

AF ablation in patients with HF provides greater benefit than the use of antiarrhythmic medications due to higher sinus rhythm maintenance rate, improved functional capacity and quality of life, improved NYHA class, longer 6-minute-walk distance, improved peak VO_2_, ^9^ reduced biomarker (brain natriuretic peptide [BNP]) levels, increased ejection fraction, ^9^ ^,^ ^10^ and reduced HF hospitalization, HF death or hospitalization, and death from any cause. ^9^ ^–^ ^11^ However, the success rate ranges from 60 to 80% at 1 year, when structural heart disease is a risk factor for recurrence. ^12^ Pulmonary-vein isolation can be achieved by radiofrequency or cryoablation, and these techniques can be combined with ablation of other substrates. Benefits include symptom control in patients with paroxysmal/persistent AF and the promotion of reverse remodeling in patients with ventricular dysfunction due to AF-induced tachycardiomyopathy, regardless of symptoms.

## New Forms of Cardiac Pacing in HF

Ventricular pacing through the native His-Purkinje conduction system may be an option for patients with pacemaker indication, given the deleterious effects of isolated right ventricular (RV) pacing in patients with HF. ^13^ Small trials suggest that His bundle pacing may result in a decrease in the incidence of cardiomyopathy, reduction in the combined endpoint of hospitalization or death, and improvement in LV dimensions and HF symptoms compared to isolated RV pacing. ^13^ ^,^ ^14^ The American guideline for the management of bradycardia recommends His bundle pacing or cardiac resynchronization therapy for patients with ventricular dysfunction who have atrioventricular block with an indication for permanent pacemaker instead of isolated RV pacing. ^15^ Compared to cardiac resynchronization therapy, His bundle pacing had an equivalent effect, with a significant reduction in QRS duration and improvement in LVEF, HF symptoms, and quality of life. ^16^ ^,^ ^17^ His bundle pacing was also tested in patients with HFrEF and right bundle branch block, leading to increased LVEF and narrowing of QRS duration. ^18^

## Percutaneous Treatment of Tricuspid Regurgitation in HF

The advent of percutaneous treatment of functional tricuspid regurgitation is attractive in selected patients at high surgical risk, for an expected improvement of symptoms. Patients who may benefit from this treatment include those with HF refractory to optimized medical therapy or with early signs of RV dysfunction, who are considered at high risk for conventional cardiac surgery or inoperable. Devices are divided into annuloplasty systems, tricuspid valve repair systems, and prosthetic valves for vena cava stenting. Although safety and efficacy clinical trials appear to be promising, the available evidence is based on single-center observational studies or registries. Therefore, more robust evidence is needed before we can confidently indicate any treatment. ^19^

## List of Participants of the Heart Failure Summit Brazil. 2020 / Heart Failure Department - Brazilian Society of Cardiology

Aguinaldo Freitas Junior, Andréia Biolo, Antonio Carlos Pereira Barretto, Antônio Lagoeiro Jorge, Bruno Biselli. Carlos Eduardo Montenegro, Denilson Campos de Albuquerque, Dirceu Rodrigues de Almeida, Edimar Alcides Bocchi, Edval Gomes dos Santos Júnior, Estêvão Lanna Figueiredo, Evandro Tinoco Mesquita, Fabiana G. Marcondes-Braga, Fábio Fernandes, Fabio Serra Silveira, Felix José Alvarez Ramires, Fernando Atik, Fernando Bacal, Flávio de Souza Brito, Germano Emilio Conceição Souza, Gustavo Calado de Aguiar Ribeiro, Humberto Villacorta Jr., Jefferson Luis Vieira, João David de Souza Neto, João Manoel Rossi Neto, José Albuquerque de Figueiredo Neto, Lídia Ana Zytynski Moura, Livia Adams Goldraich, Luís Beck-da-Silva Neto, Luís Eduardo Paim Rohde, Luiz Claudio Danzmann, Manoel Fernandes Canesin, Marcelo Bittencourt, Marcelo Westerlund Montera, Marcely Gimenes Bonatto, Marcus Vinicius Simões, Maria da Consolação Vieira Moreira, Miguel Morita Fernandes da Silva, Monica Samuel Avila, Mucio Tavares de Oliveira Junior, Nadine Clausell, Odilson Marcos Silvestre, Otavio Rizzi Coelho Filho, Pedro Vellosa Schwartzmann, Reinaldo Bulgarelli Bestetti, Ricardo Mourilhe Rocha, Sabrina Bernadez Pereira, Salvador Rassi, Sandrigo Mangini, Silvia Marinho Martins, Silvia Moreira Ayub Ferreira, Victor Sarli Issa.
